# One-step preparation of deep eutectic solvents/ reduced graphene oxide composite materials for the removal of dibenzothiophene in fuel oil

**DOI:** 10.1038/s41598-023-28041-0

**Published:** 2023-01-16

**Authors:** Yue Liu, Xiaoping Su, Yingna Cui, Xin Zhou

**Affiliations:** Department of Environmental and Chemical Engineering, Dalian University, Dalian, China

**Keywords:** Environmental chemistry, Environmental sciences, Chemistry, Environmental chemistry, Green chemistry, Materials chemistry

## Abstract

Deep eutectic solvents (DES)/reduced graphene oxide (rGO) composite materials can remove dibenzothiophene (DBT) from fuel oil by adsorption desulfurization. However, the whole synthesis process is complicated, and the DES/rGO is high-cost and not strong enough, which limits its application in industry. Therefore, a one-step method to produce DES/rGO composite materials is proposed. The performance of desulfurization was studied and the feasibility of industrial application was analyzed. First, the proposed method can improve the single extraction efficiency to 94.8% without a complicated process. Secondly, the DES/rGO can also be reused and recycled. Third, the proposed method overcomes the inconvenient storage and transportation of liquid DES. Finally, the proposed method could effectively reduce the cost of desulphurization in the industry.

## Introduction

In recent years, the frequent occurrence of haze weather not only leads to more people suffering from respiratory diseases, cardiovascular and cerebrovascular diseases but also increases the risk of cancer. One key factor leading to haze weather is the use of fuel oil as there are sulfides such as dibenzothiophene (DBT), elemental sulfur, and mercaptans in it. The presence of these sulfides makes the fuel oil produce harmful gases such as sulfur dioxide after combustion. The emission of sulfur dioxide can increase the concentration of particulate matter 2.5 (PM 2.5), which can not only cause environmental pollution but also endanger human health^1–4^. Therefore, it is necessary to remove sulfides from the fuel.

At present, the desulfurization technologies applied in industry include hydrodesulfurization^5–8^, oxidative desulfurization^9^, extractive desulfurization^10–12^, adsorption desulfurization^13,14^. Among them, hydrodesulfurization needs to be carried out under the high requirements of reaction conditions, such as high temperature, ultra-high pressure, and the presence of hydrogen. In addition, the catalyst needed in the reaction process is easy to be poisoned and inactivated. Extractive desulfurization, oxidative desulfurization and adsorption desulfurization are non-hydrodesulfurization technologies. The desulfurization conditions required by those are relatively mild. Ionic liquids (ILs) have a good ability to extract organic sulfides; however, complicated preparation steps, high cost, and large pollution of raw materials occur^15–17^. Similar to ILs, deep eutectic solvents (DES) can effectively remove sulfides without subsequent treatment of the product and simple preparation process^18–20^. In fact, the DES are not conducive to transportation and storage as they are liquid. Liu et al. immobilized DES on reduced graphene oxide (rGO)^21^. However, the cost of rGO required for the entire composite improvement process is high. In addition, the whole process of preparing composite materials is cumbersome because of the complex preparation steps of the entire composite materials including the preparation of the DES, the reduction of graphene oxide (GO) with vitamin C (VC), and the immobilization of DES on the rGO. Last but not least, the single physical impregnation method for solidification makes the combination of DES and rGO not strong enough.

In this paper, a one-step preparation of DES/rGO composite materials was achieved. Specifically, VC was added to DES, and in the process of VC reducing GO, the combination of rGO and DES was realized. GO can be more firmly bonded with the DES through chemical bonds during the reduction process via the functional groups such as carboxyl and hydroxyl groups. The proposed process not only simplifies the preparation steps of the composite materials but also enables the composite materials to bond more firmly. Composite materials of different components and different proportions were explored. The extraction efficiency of DBT from composite materials under different desulfurization conditions was studied. The optimum desulfurization conditions of the composite materials were obtained.

## Experimental

### Material

#### Characterization

The gas chromatograph is G-3900, purchased from Tianmei Technology Co., Ltd. Thermogravimetric analysis is STA6000, purchased from Perkin-Elmer, USA, and FT-IR is Nicolet is20, purchased from Thermo Fisher Scientific (China) Co., Ltd.

### Preparation of GO

GO was prepared from natural graphite powder by the improved Hummers method. First, 70 mL of 98% H_2_SO_4_ and 1.5 g of NaNO_3_ were added to the beaker. Then, under ice bath conditions, when the temperature was lower than 5 °C, 3 g of natural graphite powder was added under stirring conditions. After stirring evenly, 9 g of KMnO_4_ was slowly added, and the reaction temperature during this process should not exceed 20 °C. After reacting at 40 °C for 2 h, 150 mL of deionized water was added. After 15 min of stirring at 95 °C, 500 mL of deionized water was added. The reaction was terminated by adding 10 mL H_2_O_2_ 30%. At this point, it can be observed that the color of the solution changes from brown-black to bright yellow. Next, the product was washed by adding 100 mL of diluted HCl aqueous solution (1:10). Finally, the product was washed with deionized water until neutral, and then freeze-dried for later use.

### Preparation of DES/rGO

**Figure 1:**
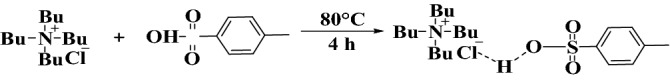
The preparation of TBAC/ p-TsOH.

The DES are mainly composed of hydrogen bond donor (HBD) and hydrogen bond acceptor (HBA). In addition, the extranuclear electron arrangement of Fe^3+^ allows Fe^3+^ to easily interact with the π electrons of DBT to facilitate desulfurization, so FeCl_3_ is often added to DES. In this section, take DES prepared by tetrabutylammonium chloride (TBAC) and paratoluenesulfonic acid (p-TsOH) as an example. The preparation route is shown in Fig. 1. First, GO dispersion is obtained by adding 10 mL deionized water to 0.01 g GO for 1 h ultrasound. 5 g of TBAC and p-TsOH with a molar ratio of 1:2 and 0.1 g of VC are stirred at 80 °C for 4 h, and then GO dispersion is added. Finally, the mixture is stirred at 95 °C for 13 h. Choline chloride (ChCl), tetramethylammonium chloride (TMAC), tetraethylammonium chloride (TEAC), propionic acid (Pr), 5- sulfosalicylic acid (SSA) and polyethylene glycol 400 (PEG) were also used to prepare DES. TBAC/2p-TsOH, 4TBAC/PEG/0.05FeCl_3_, TEAC/2p-TsOH, TMAC/2p-TsOH, ChCl/2p-TsOH/0.5Pr, ChCl/2p-TsOH, ChCl/p-TsOH and ChCl/2SSA were prepared. They are all homogeneous, a thick liquid at room temperature. They were loaded on rGO respectively to obtain eight DES/rGO composite materials. The mass fraction purity and CAS registration number of chemicals required for composite preparation are shown in Table 1. The preparation conditions of two-component and three-component DES are shown in Table 2. Water content is determined by weighing the mass of the composite materials before and after the removal of water.

**Table 1:** CAS registry number, mass fraction of the chemicals.

Component	CAS Reg.No	Suppliers	Mass fraction
Choline chloride	67-48-1	Shanghai Aladdin Industrial Corporation	98%
p-Toluene sulfonic acid-hydrate	6192-52-5	Shanghai Aladdin Industrial Corporation	≥ 98.5%
Dibenzothiophene	132-65-0	Shanghai Aladdin Industrial Corporation	≥ 99%
Ferric chloride	7705-08-0	Shanghai Aladdin Industrial Corporation	≥ 99.9%
Periodic acid	10450-60-9	Shanghai Aladdin Industrial Corporation	≥ 99.0%
Cyclohexanone peroxide	12262-58-7	Shanghai Aladdin Industrial Corporation	50%
Tetramethylammonium chloride	75-57-0	Shanghai Aladdin Industrial Corporation	≥ 98.0%
Tert butyl hydroperoxide	75-91-2	Shanghai Aladdin Industrial Corporation	70%
30% Hydrogen peroxide	7722-84-1	Tianjin Kemeiou Chemical Reagent Co., Ltd	30%
5-Sulfosalicylic acid	5965-83-3	Tianjin Kemeiou Chemical Reagent Co., Ltd	≥ 99.0%
Concentrated sulfuric acid	7664-93-9	Tianjin Kemeiou Chemical Reagent Co., Ltd	98%
Muriatic acid	7647-01-0	Tianjin Kemeiou Chemical Reagent Co., Ltd	37%
n-Octane	111-65-9	Tianjin Damao Chemical Reagent Factory	95%
Potassium permanganate	89-04-01	the No. 1 branch of Dandong Chemical Plant No. 2	≥ 99.5%
Polyethylene glycol 400	25322-68-3	Sinopharm Chemical Reagent Co., Ltd	CP
Methyl tert-butyl ether	1634-04-4	Sinopharm Chemical Reagent Co., Ltd	≥ 99.0%
Tetrabutylammonium chloride	1112-67-0	Shanghai Titan Scientific Co., Ltd	95%
Vitamin c	50-81-7	Nanjing Dulai Biotechnology Co., Ltd	≥ 99.0%
n-Hexadecane	544-76-3	Shanghai Aladdin Biochemical Technology Co., Ltd	98%
Sodium nitrate	7631-99-4	Shanghai Aladdin Biochemical Technology Co., Ltd	99.0%
Propionic acid	79-09-4	Shanghai Aladdin Chemistry Co., Ltd	≥ 99.5%
Tetraethylammonium chloride	56-34-8	Shanghai Macklin Biochemical Co., Ltd	98%

**Table 2:** Preparation of DES.

Serial number	HBA	HBD	The third compound	The molar ratio of HBA:HBD:The third compound	Preparation temperature/ ℃	Water content (%)
1	TBAC	p-TsOH		1:2	80	8.3
2	TBAC	PEG	FeCl_3_	4:1:0.05	60	5.3
3	TEAC	p-TsOH		1:2	80	5.3
4	TMAC	p-TsOH		1:2	80	6.2
5	ChCl	p-TsOH	Pr	1:2:0.5	80	6.7
6	ChCl	p-TsOH		1:2	80	1.1
7	ChCl	p-TsOH		1:1	80	6.1
8	ChCl	SSA		1:2	120	1.7

### Analytical method

For the determination of sulfur content in simulated oil, gas chromatography is mainly used in this paper, and the chromatography conditions are as follows: HP-5 column; injection volume: 0.06 μL; carrier gas (N_2_): 210 mL/min; H_2_: 40 mL/min; air: 350 mL/min; flow rate: 1.6 mL/min, constant flow mode; split ratio: 20:1; column temperature: 200 °C; inlet temperature: 270 °C; detector temperature: 270 °C, start time 3 min. The concentration of DBT was determined by the internal standard method, and 2000 mg/L of n-hexadecane was used as the internal standard to obtain a standard curve with a correlation coefficient of 0.99959. The extraction efficiency (EE) of the composite materials is obtained by measuring the content of each component in the simulated oil by gas phase, as shown in Eq. (1).

1 $$EE = \frac{{\left( {Ci - Cf} \right)}}{Ci} \times 100\%$$

$Ci$ is the sulfur content in the fuel before desulfurization, and $Cf$ is the sulfur content in the fuel after desulfurization. All the experiments in this study were done in triplicates to determine reproducibility, and the experimental errors were within 3%.

## Results and discussion

### The effect of oxidant on extraction efficiency

**Figure 2:**
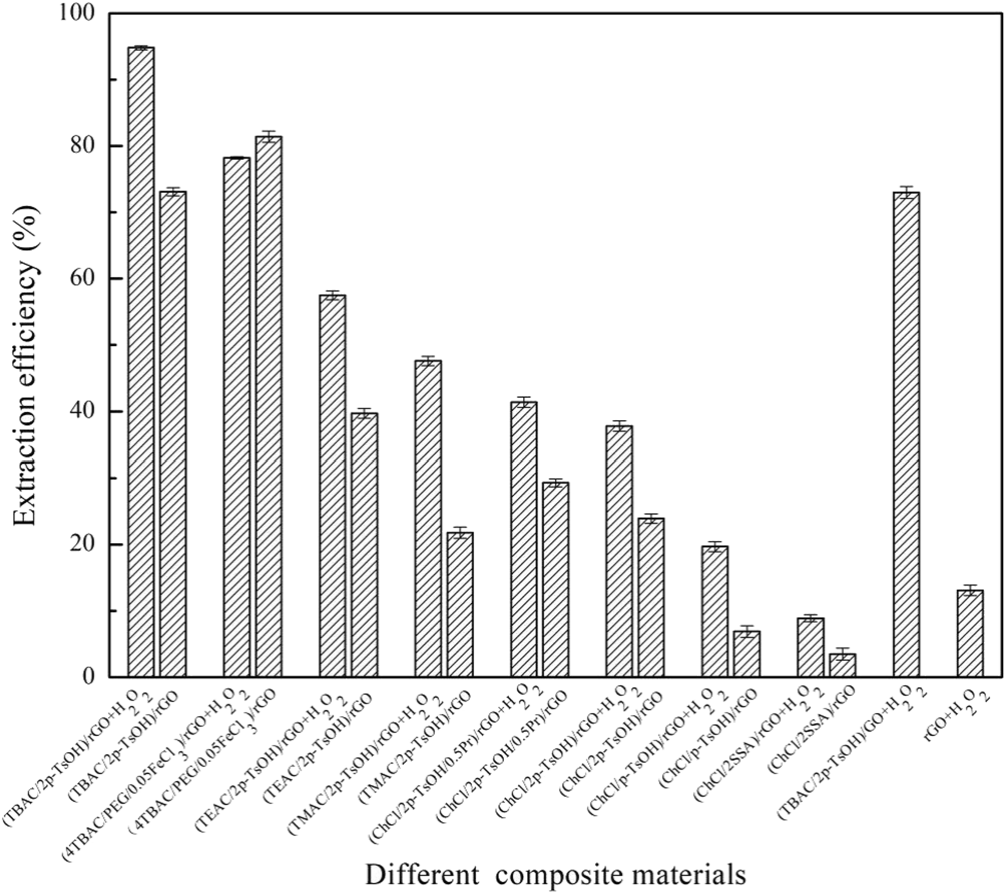
The effect of adding oxidant on extraction efficiency (experimental conditions: 1.2 g composite material, 0.5 g simulated oil (1600 mg/L sulphur content), 25 °C, 800 rpm, 60 min).

**Figure 3:**
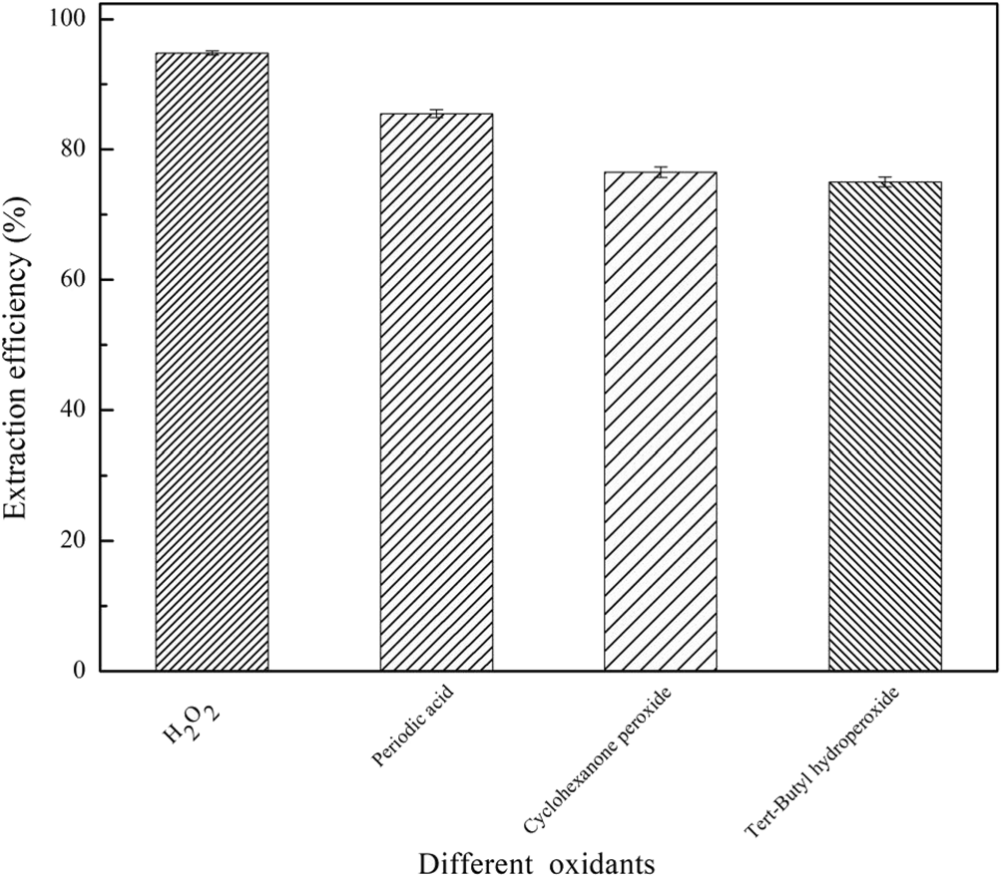
The effect of different oxidants on the extraction efficiency (experimental conditions: 1.2 g (TBAC/2p-TsOH)/rGO, 0.5 g simulated oil (1600 mg/L sulphur content), 25 °C, 800 rpm, 60 min).

**Figure 4:**
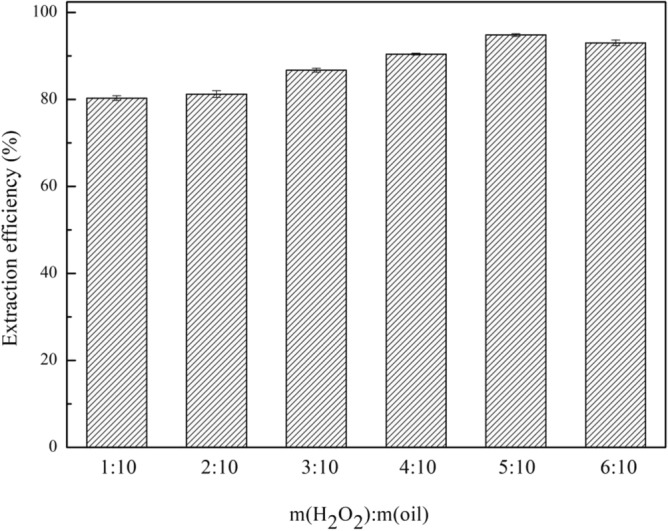
The effect of oxidant amount on the extraction efficiency (experimental conditions: 1.2 g (TBAC/2p-TsOH)/rGO, 0.5 g simulated oil (1600 mg/L sulphur content), 25 °C, 800 rpm, 60 min).

In this section, the eight composite materials were prepared to study the effect of the oxidant on the extraction efficiency during the desulfurization process. The experimental results are presented in Fig. 2. The extraction efficiency of these composite materials with H_2_O_2_ (m(H_2_O_2_): m(oil) = 1:2) is higher than that without H_2_O_2_ except (4TBAC/PEG/0.05FeCl_3_)/rGO. Because H_2_O_2_ can oxidize DBT into sulfone with a higher polarity and easily dissolve in polar composite materials^22^. As a result, the addition of an oxidant is beneficial for improving the extraction efficiency of composite materials. For (4TBAC/PEG/0.05FeCl_3_)/rGO, metalions in (4TBAC/PEG/0.05FeCl_3_)/rGO act as coordination compounds for desulfurization^23^. Therefore, (4TBAC/PEG/0.05FeCl_3_)/rGO is different from the other seven composite materials. In the following experiment, (4TBAC/PEG/0.05FeCl_3_)/rGO was extracted without H_2_O_2_. When the DES is TBAC/2p-TsOH, the curing degree and extraction efficiency of the composite materials are the highest, and the single extraction efficiency is 94.8%. And we also prepared (TBAC/2p-TsOH)/GO to explore the extraction efficiency of GO compounded with DES without reduction. It is found that, the extraction efficiency of (TBAC/2p-TsOH)/GO is lower than that of (TBAC/2p-TsOH)/rGO under the same conditions. Compared with rGO, GO surface contains more carboxyl groups, hydroxyl groups and other functional groups, which leads to the agglomeration of GO. Therefore, the adsorption sites during desulfurization also decrease, resulting in the decrease of extraction efficiency. This also confirms the necessity of reducing GO in the process of preparing composite materials. Moreover, the single extraction efficiency of (TBAC/2p-TsOH)/rGO is higher than that of rGO. In addition, we also compared the effect of four oxidants on the extraction efficiency of (TBAC/2p-TsOH)/rGO. The four oxidants are H_2_O_2_, periodic acid, cyclohexanone peroxide, and tert-butanol peroxide. As shown in Fig. 3, the extraction efficiency, of H_2_O_2_ as oxidant at 94.8% is the highest. In fact, with non-toxic and non-polluting properties, H_2_O_2_ can not introduce impurities into the system. Therefore, we use H_2_O_2_ as the oxidant for desulfurization. In addition, given that the amount of H_2_O_2_ is one of the factors affecting the extraction efficiency. It can be seen in Fig. 4, as m(H_2_O_2_):m(oil) gradually increases from 1:10 to 5:10, the extraction efficiency synchronous increases gradually. When m(H_2_O_2_): m(oil) = 5:10, the extraction efficiency is the highest, and as m(H_2_O_2_):m(oil) was increased to 6:10, the desulfurization rate does not continue to increase. At this time, there is a competitive reaction between the DBT oxidation reaction of hydrogen peroxide as an oxidant and the self-decomposition reaction of hydrogen peroxide, resulting in a decrease in extraction efficiency. When the content of hydrogen peroxide is too high, the water produced by oxidation and the water produced by the self-decomposition of hydrogen peroxide hinder the catalytic oxidative desulfurization of the system^21^. Therefore, the optimal mass ratio of m(H_2_O_2_):m(oil) is 5:10.

### Influence of mass ratio of DES to rGO on extraction efficiency

**Figure 5:**
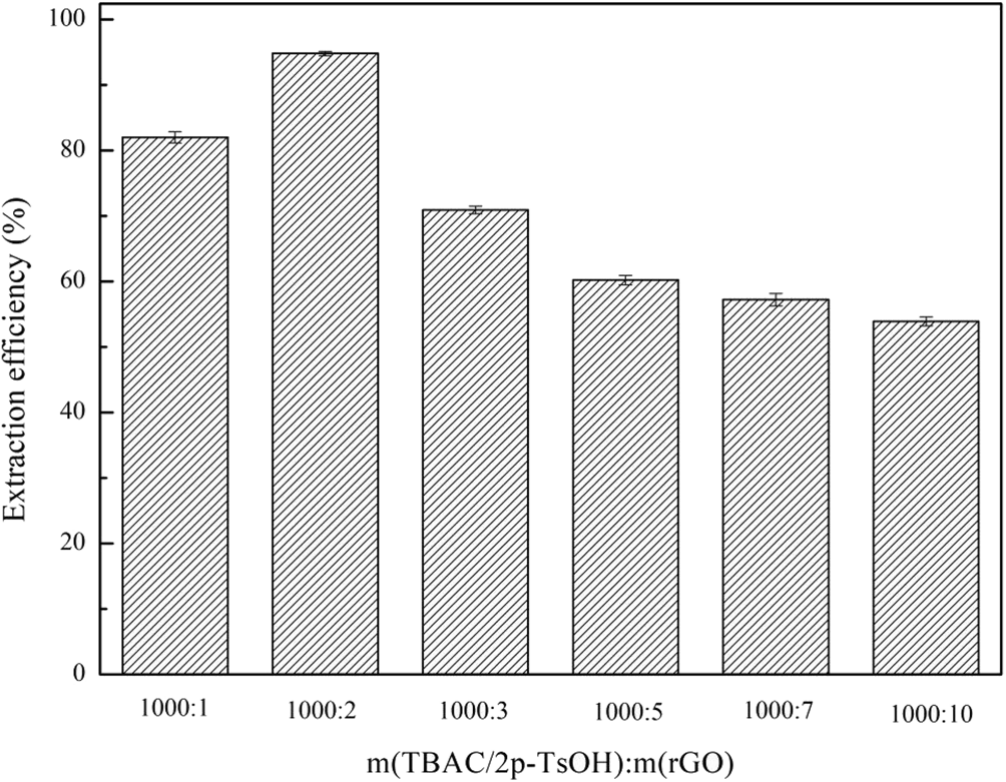
The effect of different loading amounts of TBAC/2p-TsOH on the extraction efficiency (experimental conditions: 1.2 g (TBAC/2p-TsOH)/rGO, 0.5 g simulated oil (1600 mg/L sulphur content), 0.25 g H_2_O_2_, 25 °C, 800 rpm, 60 min).

To reduce GO, different amount of VC were added during the preparation of DES. Meanwhile, when the DES is TBAC/2p-TsOH, the obtained composite materials were subjected to desulfurization experiments to explore the optimal mass ratio of DES to rGO. As shown in Fig. 5, within a certain range, as the proportion of rGO increases, the extraction efficiency of the composite material also increases. When m(TBAC/2p-TsOH):m(rGO) = 1000:2, the extraction efficiency is as high as 94.8%. Because the rGO takes advantage of the large specific surface area during the desulfurization process, which provides adsorption sites for desulfurization after compounding with DES. Continuing to increase the proportion of rGO, the desulfurization of the composite materials showes a downward trend. Because as the proportion of rGO increases, the amount of VC required in the system also increases proportionally, and VC itself does not have an obvious desulfurization effect. Therefore, we choose m(TBAC/2p-TsOH):m(rGO) = 1000:2 as the best mass ratio in follow-up experiments.

### Effect of composite materials amount on extraction efficiency

**Figure 6:**
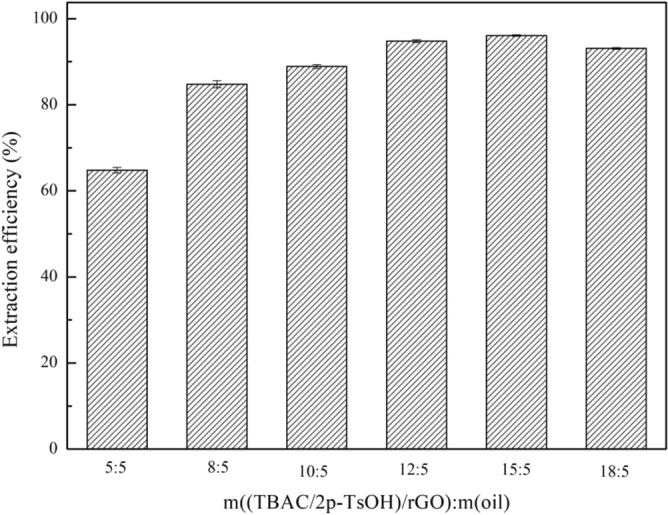
The effect of the amount of composite materials on the extraction efficiency (experimental conditions: 1600 mg/L sulphur content, 0.25 g H_2_O_2_, 25 °C, 800 rpm, 60 min).

In this section, the optimal mass ratio of composite materials to oil was explored by removing DBT with a sulfur content of 1600 mg/L in 0.5 g of simulated oil, using different amounts of composite materials, under the conditions of 25 °C, 800 rpm, 0.25 g H_2_O_2_. From Fig. 6, with the increase of the amount of composite materials, the extraction efficiency gradually increases. Because the increase in the amount of composite materials increases the number of active sites in the entire desulfurization system, which can effectively remove more DBT. When m((TBAC/2p-TsOH)/rGO):m(oil) is 12:5, the extraction efficiency is as high as 94.8%. Continuing to increase the proportion, the changing trend of extraction efficiency tends to be decreased, indicating that the removal of DBT has reached the limit. It shows that too many composite materials cannot improve the extraction efficiency, but can bring higher desulfurization costs. Therefore, when m((TBAC/2p-TsOH)/rGO):m(oil) is 12:5, it is the best mass ratio, and subsequent experiments can explore based on this ratio.

### The effect of temperature on extraction efficiency

**Figure 7:**
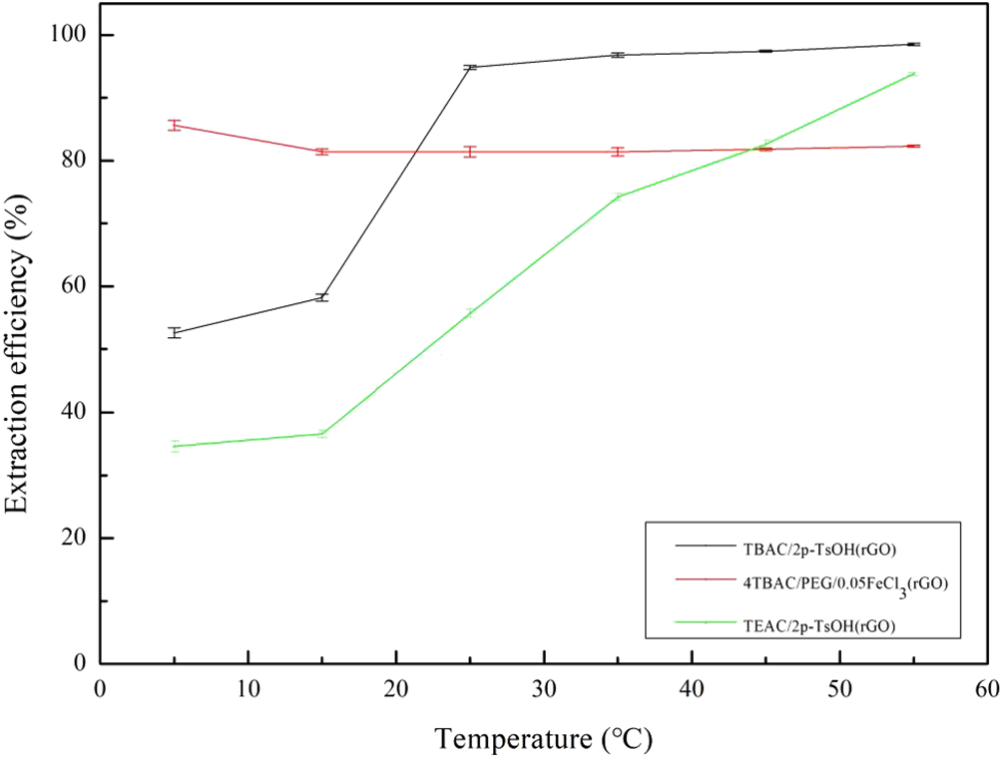
The effect of system temperature on the extraction efficiency (experimental conditions: 1.2 g composite material, 0.5 g simulated oil (1600 mg/L sulphur content), 800 rpm, 60 min).

To explore the effect of temperature on the extraction efficiency of the composite materials, 1.2 g (TBAC/2p-TsOH)/rGO, (4TBAC/PEG/0.05FeCl_3_)/rGO, and (TEAC/2p-TsOH)/rGO composite materials were used, respectively. Under the conditions of 800 rpm and 60 min, by changing the reaction temperature to 5 °C, 15 °C, 25 °C, 35 °C, 45 °C, 55 °C, DBT with a sulfur content of 1600 mg/L in 0.5 g of simulated oil was removed. As shown in Fig. 7, for (TBAC/2p-TsOH)/rGO, between 5 °C and 25 °C, the extraction efficiency increases gradually with the increase of temperature. The composite materials can achieve extraction efficiency of 94.8% at 25 °C. Between 25 °C and 55 °C, the increasing trend of the extraction efficiency gradually flattened as the temperature increase. For (TEAC/2p-TsOH)/rGO, between 5 °C and 55 °C, the extraction efficiency has always shown a large upward trend with the increase of temperature, indicating that the extraction efficiency of composite materials is greatly affected by temperature. Because with the increase of temperature, the movement of molecules is accelerated, the probability of molecular collision is increased, and the extraction efficiency is improved. For (4TBAC/PEG/0.05FeCl_3_)/rGO, between 5 °C and 55 °C, (4TBAC/PEG/0.05FeCl_3_)/rGO is stable to temperature change, and the maximum extraction efficiency of composite materials can be achieved at low temperature. This shows that different composite materials have their own characteristics. (4TBAC/PEG/0.05FeCl_3_)/rGO has reached its extraction limit and has no room to rise. In conclusion, (TBAC/2p-TsOH)/rGO can achieve a higher extraction efficiency at a relatively mild temperature compared to the other two composite materials. Considering the simplicity of experimental conditions, we set the room temperature at 25 °C as the temperature condition for subsequent experiments.

### The effect of time on extraction efficiency

**Figure 8:**
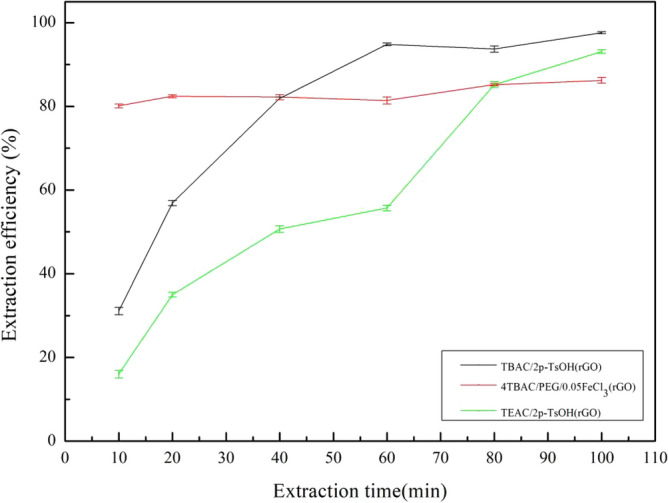
The effect of extraction time on the extraction efficiency (experimental conditions: 1.2 g composite material, 0.5 g simulated oil (1600 mg/L sulphur content), 25 °C, 800 rpm).

1.2 g (TBAC/2p-TsOH)/rGO, (4TBAC/PEG/0.05FeCl_3_)/rGO, and (TEAC/2p-TsOH)/rGO composite materials were used to explore the effect of time on the extraction efficiency of the composite materials. Under the conditions of 25 °C and 800 rpm, DBT with a sulfur content of 1600 mg/L in 0.5 g simulated oil was removed by changing the reaction time from 10 to 100 min. The experimental results are shown in Fig. 8. For (TBAC/2p-TsOH)/rGO and (TEAC/2p-TsOH)/rGO, the extraction efficiency increases gradually with the increase of reaction time. But after 60 min, the trend of extraction efficiency remained flat. Due to sufficient reaction time, the composite materials, DBT and H_2_O_2_ can be fully contacted to achieve desulfurization. The amount of H_2_O_2_ in the system decreases, and the extraction efficiency can not be improved by continuing to prolong the reaction time. For (4TBAC/PEG/0.05FeCl_3_)/rGO, the extraction efficiency can not change much with the increase of reaction time as there is no H_2_O_2_ in the reaction system of such composite materials. In summary, (TBAC/2p-TsOH)/rGO can achieve a higher extraction efficiency in a relatively shorter reaction time than the other two composite materials. Considering the time cost of the experiment, we took 60 min as the time condition for the subsequent experiments.

### Influence of rotational speed on extraction efficiency

**Figure 9:**
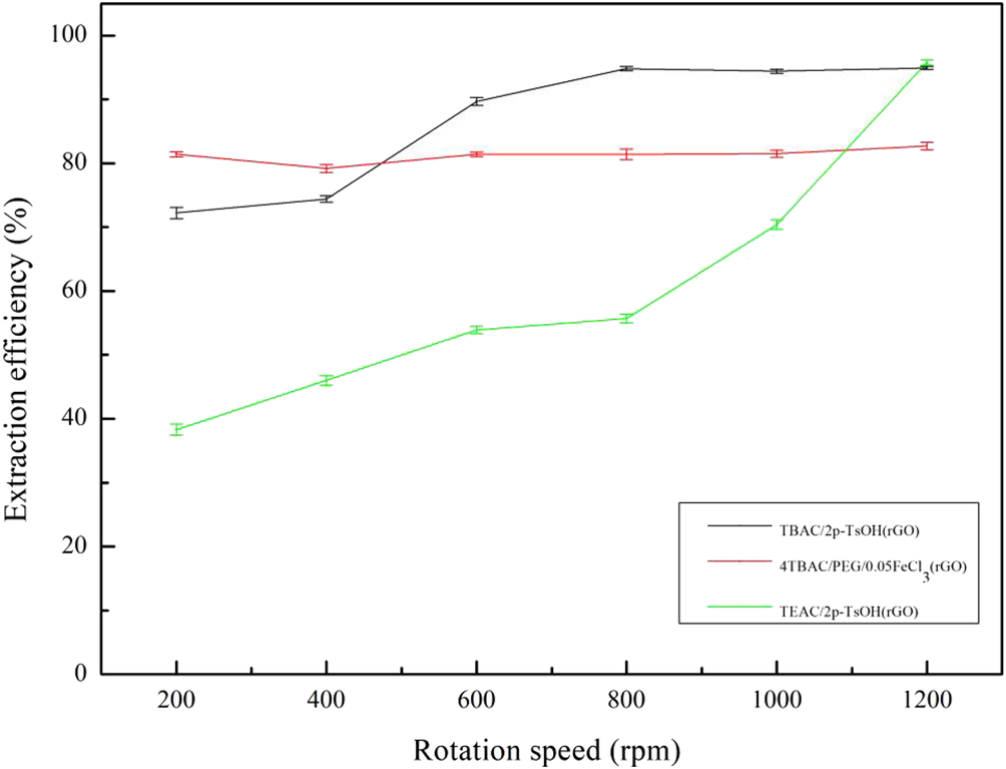
The effect of rotation speed on the extraction efficiency (experimental conditions: 1.2 g composite material, 0.5 g simulated oil (1600 mg/L sulphur content), 25 °C, 60 min).

1.2 g (TBAC/2p-TsOH)/rGO, (4TBAC/PEG/0.05FeCl_3_)/rGO, and (TEAC/2p-TsOH)/rGO composite materials were used to explore the effect of rotational speed on the extraction efficiency of the composite materials. Under the conditions of 25 °C and 60 min of reaction, DBT with a sulfur content of 1600 mg/L in 0.5 g of simulated oil was removed by changing the reaction speed from 200 to 1200 rpm. From Fig. 9, for (TBAC/2p-TsOH)/rGO, with the increase of the reaction speed, the extraction efficiency gradually increases before 800 rpm, and the extraction efficiency can reach up to 94.8%. After 800 rpm, with the increase of reaction speed, the extraction efficiency remained unchanged. For (TEAC/2p-TsOH)/rGO, the extraction efficiency increases gradually with the increase of reaction speed. Because with the increase of the reaction speed, the composite materials, DBT, and H_2_O_2_ can be fully contacted to achieve deep desulfurization. When the reaction speed is large enough, the amount of H_2_O_2_ in the system decreases, so the extraction efficiency is not significantly improved by continuing to increase the reaction speed. For (4TBAC/PEG/0.05FeCl_3_)/rGO, with the increase of reaction speed, the changing trend of extraction efficiency has been relatively gentle. Because there is no H_2_O_2_ in the reaction system of such composite materials, the extraction efficiency is not affected by the reduction in the amount of H_2_O_2_. In conclusion, (TBAC/2p-TsOH)/rGO can achieve a higher extraction efficiency at a relatively lower reaction speed than the other two composite materials. Considering the simplicity of the experimental conditions, we took 800 rpm as the rotational speed condition for the subsequent experiments.

### Repeated use and recycling of composite materials

**Figure 10:**
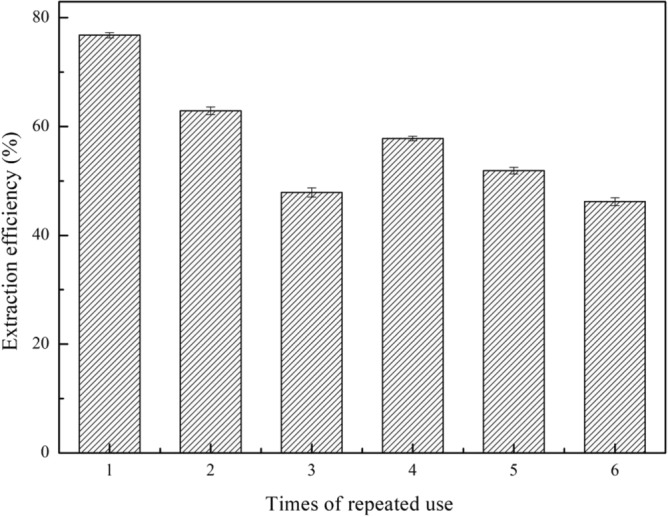
The effect of repeated use on the extraction efficiency (experimental conditions: 1.2 g (TBAC/2p-TsOH)/rGO, 0.5 g simulated oil (1600 mg/L sulphur content), 0.25 g H_2_O_2_, 25 °C, 800 rpm, 60 min).

**Figure 11:**
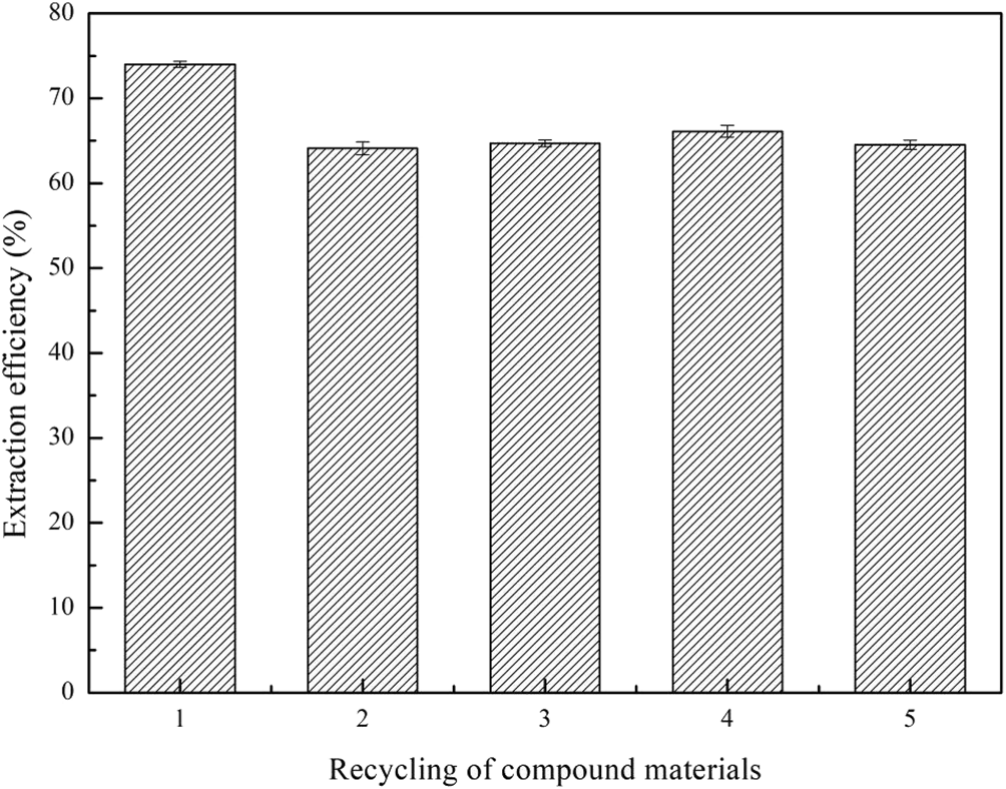
The effect of recycling on the extraction efficiency (experimental conditions: 1.2 g (TBAC/2p-TsOH)/rGO, 0.5 g simulated oil (1600 mg/L sulphur content), 0.25 g H_2_O_2_, 25 °C, 800 rpm, 60 min).

DBT was removed under the conditions of 25 °C, 800 rpm, 0.25 g H_2_O_2_, and desulfurization for 60 min, 1.2 g (TBAC/2p-TsOH)/rGO. The composite materials after desulfurization were directly used for the next desulfurization to explore the reusability of the composite materials. As shown in Fig. 10, the (TBAC/2p-TsOH)/rGO can maintain a extraction efficiency of about 50% after 6 times repeated uses. The third reuse of composite materials is clearly out of the trend. In the process of repeated use, the reduction of adsorption sites inside the composite material leads to the unstable situation of the composite materials. In addition, we also explored the recyclability of the composite materials. Through the organic solvent washing method, the desulphurized composite materials were washed with methyl tert-butyl after several times, and then dried in a vacuum drying box for use in the next desulfurization. From Fig. 11, the (TBAC/2p-TsOH)/rGO composite materials can still achieve extraction efficiency of about 60% after 5 times of recycling. In conclusion, after repeated use or recycling, the composite materials still have desulfurization performance.

### Characterization and analysis of DES/rGO composite materials

#### TGA analysis

**Figure 12:**
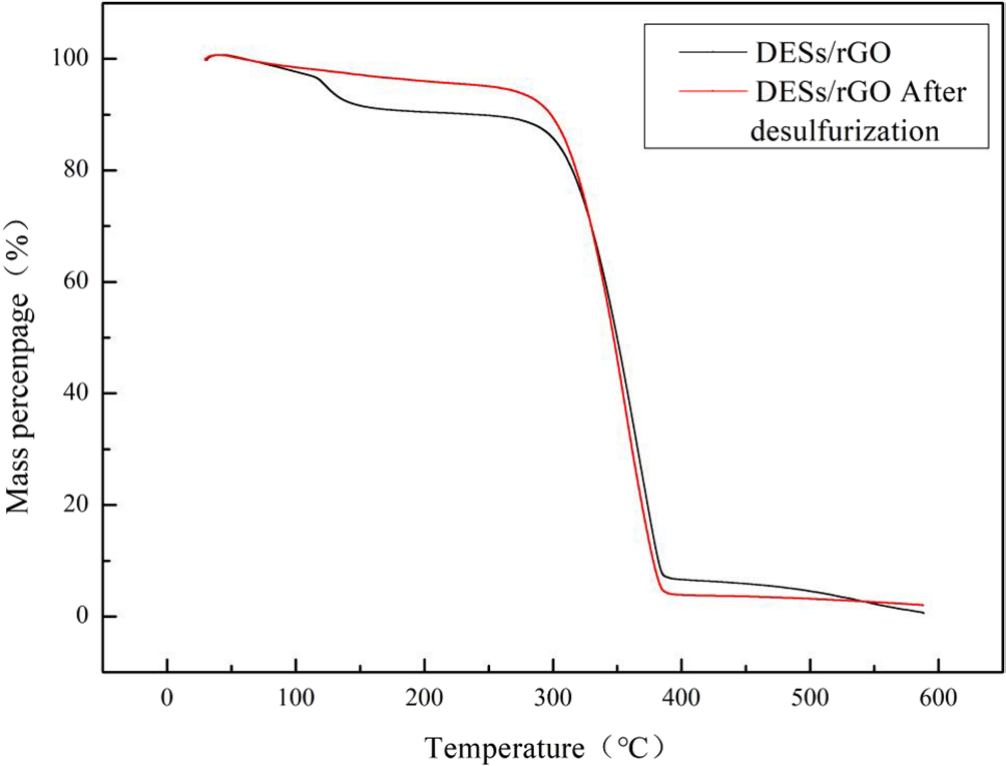
TGA curve of DES/rGO and DES/rGO after desulfurization.

In this section, thermogravimetric analysis of the composite materials before and after desulfurization was carried out to explore the thermal stability of the composite materials. Taking (TBAC/2p-TsOH)/rGO as an example, under the protection of nitrogen, the composite materials were heated from 30 to 600 °C at a heating rate of 20 °C/min. As shown in Fig. 12, at 30–150 °C, the weight of the composite materials before and after desulfurization decreased, which is the weight of water loss. Among them, the quality of the composite materials before desulfurization decreased more at 150 °C, which is the quality of VC decreased. Because there is a small amount of VC that does not participate in the reduction reaction in the composite materials before desulfurization. The H_2_O_2_ added during the desulfurization process could react with this small portion of VC. There is no VC in the desulfurized composite materials, and the thermogravimetric map produces this difference. At 300 °C, the weights of the composite materials both before and after desulfurization began to decrease significantly. It shows that at this temperature, the composite materials begin to thermally decompose, and the composite materials have good thermal stability. At 380–600 °C, the weight of the composite materials before desulfurization still decreased slightly. It shows that the composite materials are not completely thermally decomposed until nearly 600 °C. For the desulfurized composite materials, the weight remains unchanged at 380–600 °C. It shows that at 380 °C, the composite materials after desulfurization have been completely thermally decomposed. The reason for the poorer thermal stability of the composite materials after desulfurization than that before desulfurization is that the addition of hydrogen peroxide during the desulfurization process can oxidize the sulfides in the simulated oil to sulfones. The strong polarity of sulfone not only makes it better adsorbed by the composite materials, but also has a certain influence on the thermal stability of the composite materials.

#### FT-IR analysis

**Figure 13:**
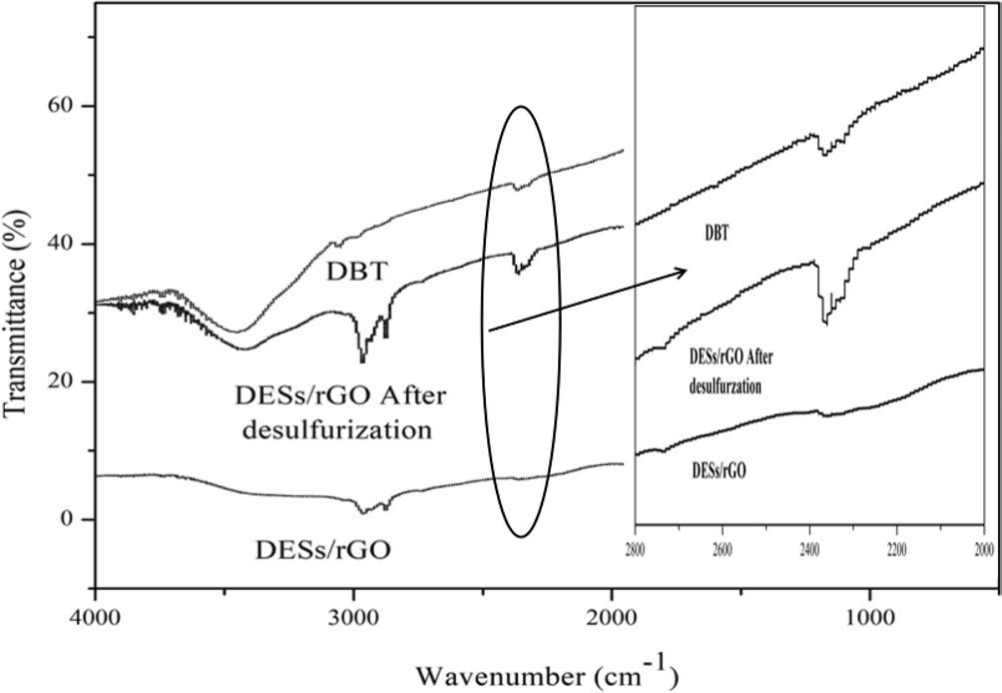
Infrared curve of DBT, DES/rGO, DES/rGO after desulfurization.

In addition, we compared the FT-IR of the composite materials before and after desulfurization by taking (TBAC/2p-TsOH)/rGO as an example. From Fig. 13, DBT has a distinct sulfur peak at 2372 cm^−1^. For the DES/rGO, there is no characteristic peak at 2372 cm^−1^ before desulfurization. But after desulfurization, there are also an obvious sulfur peak at 2372 cm^−1^. Therefore, the composite materials can effectively remove DBT from simulated oil.

## Conclusions

To remove sulfide from the fuel more effectively, we proposed a one-step method. DES with VC was prepared to reduce GO to achieve the composite of DES and rGO. Also, The optimal desulfurization conditions for (TBAC/2p-TsOH)/rGO are 25 °C, 800 rpm, m((TBAC/2p-TsOH)/rGO):m(oil):m(H_2_O_2_) = 24:10:5 for 60 min. The single extraction efficiency can reach 94.8% with lower desulfurization cost. And the desulfurized composite materials can also be reused and recycled. The composite materials after repeated reuse or recycling still have a relatively high extraction efficiency. Meanwhile, the results of the characterization of the composite materials by TGA and FT-IR show that the composite materials can effectively remove DBT from the simulated oil. The improvement not only ensures the extraction efficiency of the composite materials but also ensures the desulfurization benefit.

## Supplementary Information

Supplementary Information 1.

Supplementary Information 2.

## Data Availability

All data generated or analyzed during this study are included in this published article [and its Supplementary Information files].
