# Supplementary material for: One-step preparation of deep eutectic solvents/ reduced graphene oxide composite materials for the removal of dibenzothiophene in fuel oil
Source: Sci Rep. 2023 Jan 16;13:832. doi: 10.1038/s41598-023-28041-0 (PMC9842677; doi:10.1038/s41598-023-28041-0)
Supplement: Supplementary file 2 — Supplementary Information 2. [file 41598_2023_28041_MOESM2_ESM.pdf]

One-step preparation of deep eutectic solvents/ reduced graphene oxide composite materials for the removal of dibenzothiophene in fuel oil

Yue Liu†, Xiaoping Su†, Yingna Cui†, Xin Zhou†*

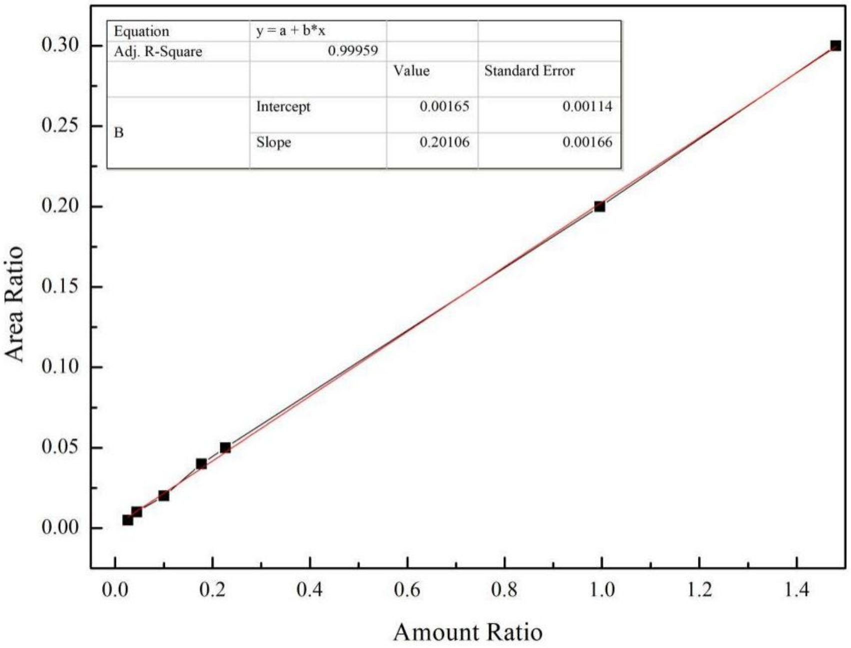

Figure 1. The standard curve of DBT
